# Standardization of Radiologic Procedures for Pediatric Videofluoroscopic Swallow Studies: A Service-based Quality Improvement Initiative

**DOI:** 10.1097/pq9.0000000000000123

**Published:** 2018-12-06

**Authors:** Benjamin Thompson, Jennifer P. Lundine, Lauren Madhoun, Houchun Hu, Dominic Holliman-Wade, D. Gregory Bates

**Affiliations:** From the * Department of Radiology, Nationwide Children’s Hospital, Columbus, Ohio; † The Department of Speech and Hearing Science, The Ohio State University, Columbus, Ohio; ‡ The Division of Clinical Therapies, Nationwide Children’s Hospital, Columbus, Ohio.

## Abstract

**Introduction:**

The videofluoroscopic swallow study (VFSS) remains the primary method to evaluate swallowing function in infants and children. However, limited standards exist to guide the image acquisition during pediatric VFSSs, leading to inadequate images, variable assessment quality, and potential for suboptimal diagnostic accuracy. The goal of this quality improvement project was to standardize the radiological procedure for radiologists at our institution.

**Methods:**

A multidisciplinary team of project leaders established 3 imaging standards to target (magnification, the field of view, and pulse repetition rate), based on the best available evidence. The team used the Institute for Healthcare Improvement’s Model for Improvement to guide the project, identified key drivers, and implemented specific interventions. Project leaders observed a total of 170 VFSS examinations, performed by radiology faculty, fellows, and residents over 24 months. The primary outcome for this project was physician compliance with 3 established imaging standards: field of view, magnification, and pulse repetition rate. Project leaders collected data without the radiologists’ knowledge, and also recorded average radiation exposure as a balancing measure during this process.

**Results:**

At baseline, compliance with all 3 criteria was 20%. Following interventions designed to improve radiologist performance with the 3 identified standards, the compliance rate reached 100% for the last 50 recorded VFSSs. During this same time, there was no meaningful increase in average radiation exposure compared with the 6 months before the implementation of this project.

**Conclusions:**

Compliance with standardized image acquisition of VFSSs can be accomplished using a systematic quality improvement protocol targeting simple, inexpensive interventions.

## Abstract 2

Supplemental Digital Content is available in the text.

## INTRODUCTION

The videofluoroscopic swallow study (VFSS) remains the primary instrumental assessment used to evaluate oropharyngeal swallowing function in infants and children dynamically.^1^ Assessment using fluoroscopy is crucial due to the high occurrence of silent aspiration for infants and children with swallowing disorders (dysphagia).^2^ A VFSS is used to define the nature and severity of underlying swallow dysfunction and to trial modifications to compensate for any identified impairment. The 2017 American College of Radiology Practice Parameter for the Performance of the Modified Barium Swallow^3^ provides a peer-reviewed guideline as to the minimum standards for technical performance for radiologists during this procedure. Interdisciplinary teams (which may include speech-language pathologists (SLPs) and/or occupational therapists (OTs), in addition to the radiologist) commonly perform VFSSs, but limited standards are available to guide the radiologist’s specific practice during a pediatric VFSS. Lack of a standardized practice guideline may lead to suboptimal examinations and interpretations with resultant inappropriate treatment plans. When professionals diagnose an infant or child with a swallowing impairment, recommendations for feeding may include disrupting breastfeeding due to the need for the provision of thickened liquids or, in some cases, non-oral feeding practices, such as nasogastric feeding tubes. Thus, it is critical that images acquired during a VFSS allow for the most accurate assessment of a child’s swallowing function.

VFSSs also require exposure to ionizing radiation. With limited guidance for standardization of radiographic techniques, nonstandardized imaging practices have the potential to increase radiation exposure for vulnerable pediatric patients. Ionizing radiation is especially damaging in the pediatric population due to the cumulative effects of radiation (stochastic effect).^4^ Adherence to the As Low As Reasonably Achievable (ALARA) principle is most important to delivering a safe and effective VFSS with the lowest radiation dose needed for diagnosis.^5^ Techniques used to reduce radiation exposure during a VFSS include collimation, limiting magnification, decreasing exposure time, and pulsed fluoroscopy.^6^

As a result of the above-stated limitations in evidence-based guidelines for standardized imaging practices related to pediatric VFSSs, the goal of this service-based quality improvement (QI) project was to standardize the radiologist’s performance during pediatric VFSSs within a tertiary pediatric referral hospital using the American College of Radiology Practice Parameter.^3^ If successful, we would publish our results using the Standards for QUality Improvement Reporting Excellence (SQUIRE) guidelines.^7^ This project did not seek to establish evidence-based guidelines, but rather to evaluate whether a group of practitioners could adopt a standard set of imaging procedures for VFSSs with infants and children. Image quality is an intrinsic property of the imaging system and is dependent on the visual perception of the observer. Spatial and temporal resolutions are 2 key features that contribute to intrinsic image quality. Spatial resolution describes the level of detail captured in an image, and temporal resolution refers to the number of images displayed over a given period.^8^ Magnification and field of view are the 2 key components of spatial resolution. Ideal temporal resolution for a VFSS is best achieved at 25–30 frames per second.^9–11^ We created guidelines related to these 3 components (magnification, the field of view, and pulse repetition rate) to standardize image collection during VFSSs.

## METHODS

### Setting and Context

In 2016, there were 6,253 fluoroscopic studies performed at the hospital and its satellite locations. Of these studies, 1,375 (22%) were VFSS. Thirteen of the 22 staff radiologists perform fluoroscopic examinations on a regular basis.

Additionally, the Department of Radiology at Nationwide Children’s has a total of 23 trainees annually. Twenty radiology residents and 3 fellows comprise the trainee group. At Nationwide Children’s Hospital, VFSSs are completed by a radiologist (staff or trainee) in conjunction with an SLP and an OT. At the time of this project, there were 12 SLPs and 8 OTs who regularly performed VFSSs. Project leaders completed the observations discussed in this article as part of this specific QI initiative. As the project was not human subject research, it did not require review and approval by the Institutional Review Board per policy.

## MEASURES

Following a review of the pertinent literature, a multidisciplinary team composed of senior SLPs, an SLP researcher, staff pediatric radiologists, and the department’s lead technologist determined the 3 key components most essential for standardizing the VFSS imaging protocol: field of view, magnification, and pulse repetition rate.

### Field of View

**Fig. 1.**
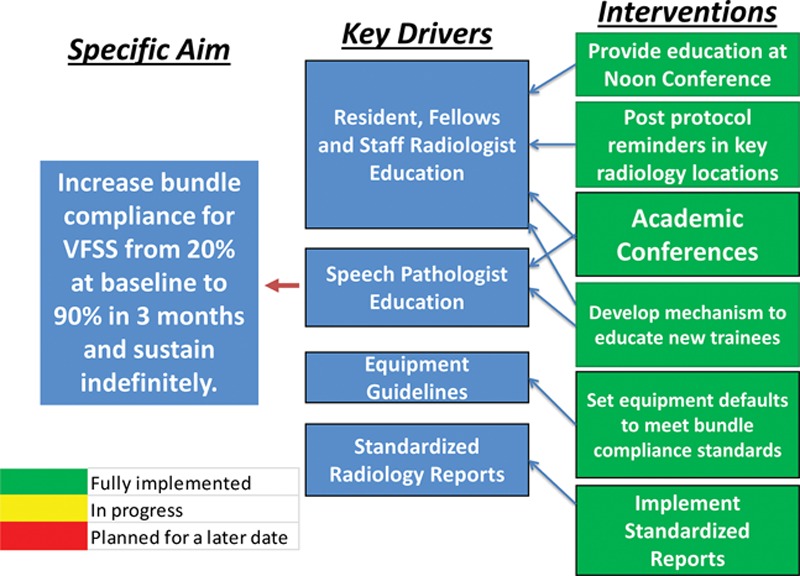
Compliance bundle requirements for VFSS.

Standardized collimation during a VFSS should provide a focused view of the oropharyngeal tract while avoiding unnecessary radiation exposure to radiosensitive organs. The superior collimation border must be below the inferior orbital rim. The inferior collimation border should be above C5 to exclude the thyroid gland. The anterior border must include the lips on at least several swallows to allow assessment of the oral phase of the swallow. The posterior collimation border should include the anterior one-third of the cervical vertebral bodies (Fig. 1). This protocol assures complete anatomical and physiological assessment of an entire oropharyngeal swallow, without focusing radiation on unnecessary anatomical landmarks.^12^

### Magnification

Due to the lack of evidence-based guidelines regarding magnification, the project team established the following protocol for our institution based on many years of experience and consultation with related professionals. Appropriate magnification is crucial to VFSS performance. Magnification must be high enough to provide anatomic detail but balanced with the ALARA principle to avoid unnecessary radiation exposure. In our experience, magnification should never exceed 2× magnifications on a standard 3× magnification scale.^12^ More specifically, practitioners should not exceed 2× magnifications in patients younger than 1 year of age and 1× magnification in children older than one.

### Pulse Repetition Rate

For VFSSs with adults, the standard of care for the pulse repetition rate has been shown to be optimal at 30 frames per second.^13^ A shorter pulse repetition rate (ie, 12.5–15 fps) is more likely to miss episodes of full-depth penetration or aspiration.^9,10^ One past study evaluating a frame rate of 25 frames per second in pediatric patients referred for a VFSS found that radiation exposure risk did not increase and that the VFSS reliably identified swallow function with a shorter radiation screening time using the higher frame rate.^11^

### Radiation Exposure

This QI project focused specifically on standardizing radiologic procedures for pediatric VFSSs. In QI projects, a balancing measure should be used to monitor unintended consequences that may occur with changes to procedures.^14^ Because radiation dosage is a critical variable during imaging studies, team members assessed average radiation exposure for VFSSs performed during the period of this project compared with average radiation exposure in the 6 months before the project’s initiation.

## KEY INTERVENTIONS

**Fig. 2.**
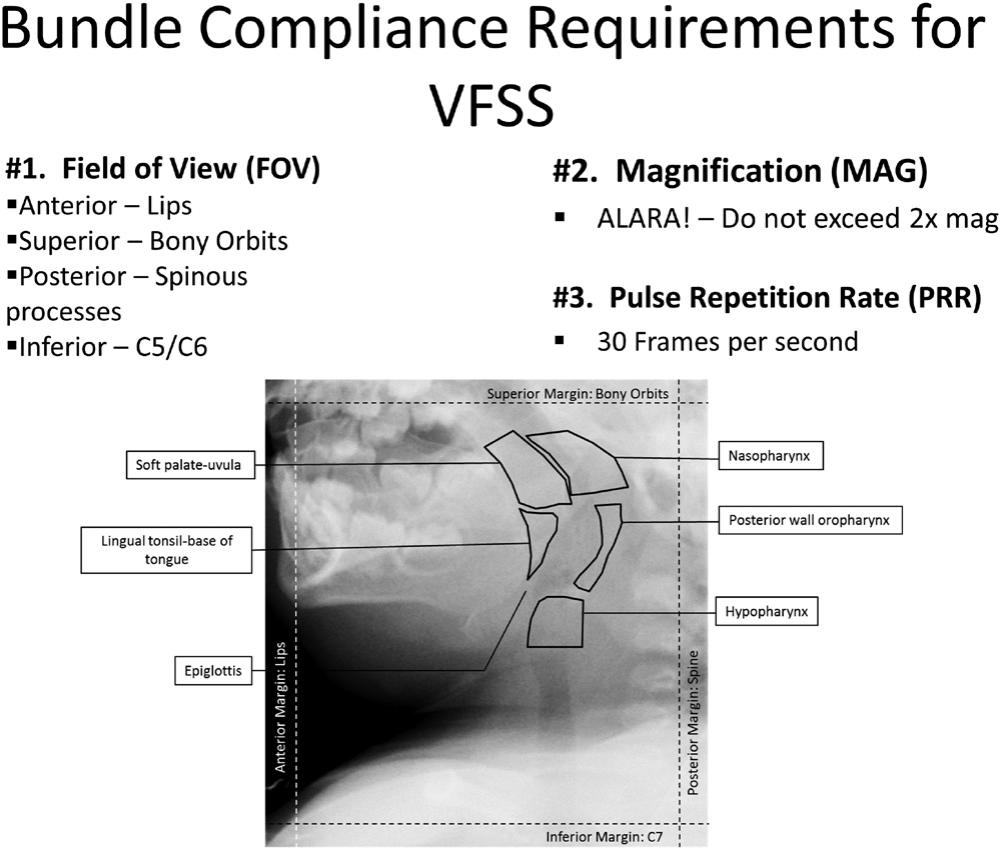
Key driver diagram for VFSS standardization.

**Fig. 3.**
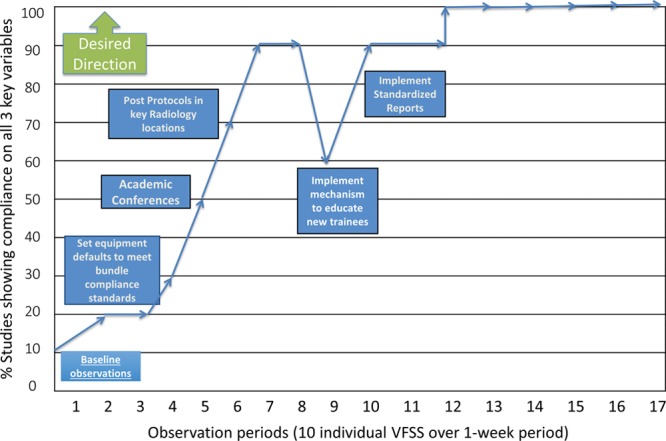
Recorded compliance with key interventions for VFSS standardization.

The multidisciplinary committee spearheading this project identified and prioritized key drivers (Fig. 2). Key drivers identified the steps needed to accomplish the goal of standardizing radiologist procedures during a VFSS. Project leaders identified the following key drivers: (1) education of residents, fellows, and staff radiologists; (2) education of SLPs; (3) equipment guidelines and default settings; and (4) standardized radiology reports. Because the goal of this project was to standardize radiologists’ performance during VFSSs, compliance with all 3 measures (magnification, field of view, and pulse repetition rate) was considered a bundle, such that radiologists must meet all 3 criterion for compliant performance. Project leaders applied interventions systematically and measured compliance after implementation of each intervention (Fig. 2). The interventions included (1) educational conferences; (2) posting of the three required standards (compliance bundle) within the fluoroscopy suite (Fig. 1); (3) implementing mechanisms to educate new trainees; (4) setting equipment defaults to comply with compliance bundle standards; and (5) implementing standardized documentation templates. Details below list the interventions found to be most impactful on radiologist performance compliance.

### Educational Conferences and Education of New Trainees

During these education sessions, a senior SLP explained the intricacies of the swallow and the requirements to perform a diagnostically accurate examination based on the best available evidence. Project leaders offered education sessions until all staff radiologists attended 1 session. To educate new trainees and maintain standards across radiologists, in addition to an in-person review of imaging guidelines by the lead technologist or a staff radiologist, all new trainees received a comprehensive packet of up-to-date literature on VFSSs. This packet included a diagram and explanation of the swallowing mechanism, and illustrations and instructions specifically explaining the compliance bundle standards. Additionally, new trainees were required to review departmental standards, pass a quiz, and demonstrate proficiency in performance before being given autonomy (**see Supplemental Digital Content** at http://links.lww.com/PQ9/A57).

### Protocol Posting Within the Fluoroscopy Suite

Protocol reminders (Fig. 1) were posted within the fluoroscopy suite so that both technologists and the radiologist performing the VFSS would see them before initiating a study.

### Standardized Radiology Reports

Project members created and implemented a concise, standardized QI pertinent template, which automatically loaded into the report dictation software (PowerScribe 360; Nuance Communications, Inc, Burlington, Mass.) used at our facility. Standardized reporting ensured that all radiologists describe each phase of the swallowing exam and not solely focus on aspiration and penetration. Radiologists within the department received this template well, and they reported that it improved uniformity and reinforced appropriate technique. Standardized reporting also allowed for continuous monitoring of our primary outcome measure during the sustainment phase.

## ANALYSIS

The primary outcome measure for this project was compliance with all 3 guidelines in the compliance bundle (magnification, field of view, and pulse repetition rate) during a VFSS (ie, compliant versus noncompliant). Project leaders considered a study compliant only when a radiologist demonstrated all 3 required standards to the guidelines established by this project. The project lead (first author) observed 20 baseline VFSSs before systematically implementing interventions. The lead completed all observations without the radiologists’ knowledge. As part of ongoing quality ratings within the department, radiology staff, and residents are aware that observers may scrutinize the quality of any examination. This practice is to assure that the department is performing quality work and that residents are meeting the expected standards of care provision.

The project lead observed VFSSs over 17 separate 1-week periods of time (Fig. 3) Project leaders defined an observational period as 10 observed VFSSs throughout a week. The lead author observed VFSSs in a separate room, which displayed the live recording and settings for the examination. The project lead observed 20 VFSSs to gather baseline data. At baseline, radiologists were performing 20% of VFSSs with compliance on the 3 required elements. The implementation of all interventions took place over a 12-month period. Following each intervention, a new set of observations was completed to document interval change in compliance with the 3 components measured for this project. Team members collected an additional 12 months of sustainability data for a total of 24 months of observational compliance.

Average radiation exposure and VFSS fluoroscopy time were recorded for the 6-months that preceded the start of this project. Once imaging compliance reached 100% for observed studies, the team assessed average radiation exposure and VFSS fluoroscopy time for the 6-month period before the implementation of this QI project.

## RESULTS

The most positive effective change noted for compliance with these new standards was following a 30-minute staff radiologist and trainee education session. Following this education, the compliance rate rose to 50%. After implementation of 3 additional interventions (ie, setting equipment defaults to meet bundle compliance standards, an additional 30-minute educational session for staff who were unable to attend the first session, and posting of protocol reminders in key radiology sections) a new standard of 90% compliance was achieved. Project leaders noted a dip in the compliance run chart to 60% following a new wave of incoming residents (Fig. 3). This degradation created an opportunity to implement a new intervention. At the start of each fluoroscopic rotation, a staff member trained the incoming residents and also required new trainees to review an educational handout explaining how to perform a VFSS. They were required to read the handout, answer questions focused on radiologist technique, and demonstrate knowledge competency before performing a VFSS. This intervention was effective, as there was an immediate rebound in the compliance rate to our new standard of 90%. Following the implementation of standardized reports, the last intervention, observations showed a 100% compliance rate for the last 50 consecutive VFSSs assessed.

Radiation exposure and average fluoroscopy time for the 6 months preceding the start of this QI project were 6.32 mGy and 2 minutes 26 seconds, respectively. With levels of compliance recorded at 100%, the average radiation dose for VFSSs was 6.12 mGy. The VFSS fluoroscopy time for that same period averaged 1 minute 57 seconds.

## DISCUSSION

The VFSS is an important tool to assess the swallowing physiology of pediatric patients with concerns of dysphagia. VFSSs often involve multiple clinicians (eg, radiologist, SLP, OT), and a standardized, evidence-based guideline for completing these examinations with infants and children is not yet available. The QI protocol presented here describes how one large pediatric teaching hospital was able to plan and implement new guidelines specifically for radiologists who lead the imaging portion of the VFSS. There are several key concepts to include when attempting to modify clinical practice: the involvement of key stakeholders, multifaceted implementation strategies, evaluation with timely feedback, and online adjustment to interventions when necessary.^15,16^ Additionally, this work is clinically relevant because it shows how radiologists can standardize the imaging procedures for VFSSs to improve the overall process. The next step in standardizing VFSSs for infants and children is the standardization of the role of the evaluating clinicians (ie, SLP and/or OT). There is a critical need for standardized techniques to guide the administration of various consistencies and the evaluation of swallowing in infants and children. Recent research has introduced a standardized assessment protocol (MBSImP, Northern Speech Services, Gaylord, Mich.)^17^ for VFSS performance in adults. Unfortunately, similar guidelines and training are not yet available for infants and children, and MBSImP does not include guidelines for radiologists. Thus, the QI project presented in this article shows that radiologists (and other invested clinicians) can standardize fluoroscopy procedures as a first step in improving the consistency of VFSSs for infants and children.

In the case of imaging for pediatric VFSSs, consistent standards are necessary to ensure appropriate visualization of oral and pharyngeal swallowing physiology, while also maintaining the safety of the patient by reducing unnecessary exposure to radiation.^11,18^ Research has shown that procedures are not consistent among radiologists, especially when comparing experienced radiologists to novice radiologists.^5^ Thus, radiologists might benefit from a structured guide for imaging during a VFSS to ensure that each study allows for optimal image collection. As described above, using a structured QI protocol, this project found that with standard equipment defaults, an educational session, posted protocol reminders, and provision of a handout that listed the guidelines (Fig. 1), observations reached 100% compliance on 3 important VFSS parameters: magnification, field of view, and pulse repetition rate. This high standard was met even with constant rotation of new resident trainees who were performing the studies.

The balance between limiting radiation exposure (ALARA principle) and performing a valid, representative VFSS is challenging. The ideal case strives to achieve the highest quality examination with the least possible radiation exposure for the patient.^11^ In cases of VFSSs, this requires that the interpreting clinician assess the patient while observing a fewer number of swallows. Thus, the radiologist must make sure that imaging is performed within a strict protocol to allow the clinician the greatest opportunity to view the swallowing pathophysiology reliably and validly. During this project, an interdisciplinary group of practitioners implemented interventions that improved the consistency of imaging procedures during pediatric VFSS, and a preliminary review of overall radiation exposure shows that radiation dosage did not increase during the period when these protocol changes were implemented compared to the 6 months before their implementation. While there are few studies of VFSSs in pediatric populations, research is beginning to show that completing VFSSs with higher frame rates appears to improve the quality of the image, thus allowing the evaluating clinician to make judgments based on fewer observations.^9–11^ Existing studies offer support to the standards chosen as part of this project. The measures described in this protocol should improve the overall image quality, allowing clinicians to view, and interpret swallowing function more quickly, while simultaneously reducing overall radiation exposure time and dosage.

An interdisciplinary group of clinicians designed this project using a QI protocol, and thus, did not have the controls that would be present in a formal research study. A limitation in this work is that radiation dosage was used as a balancing measure, and not tracked in a way that would allow for more specific statistical analyses. Future research projects should prospectively gather patient-specific data to ensure that the aggregate findings reported in this article are similar when examined on a patient-by-patient basis. While not addressed in the current project, future studies should also examine quantitative and qualitative variables related to swallowing function to ensure that these imaging guidelines do not hinder clinicians in making judgments about swallowing function.^9,11^ Additionally, because institutions use different equipment, it is difficult to detail specifications that would apply to all equipment. Consultation with technologists may be needed to determine how to set the appropriate settings for each piece of equipment.

A group of experienced practitioners from various disciplines chose the measures and their parameters for this project. Because specific evidence-based guidelines are lacking, there are modifications to these parameters that are in need of further study. For example, the literature is inconsistent regarding pulse repetition rate. Future research projects should examine these parameters to help establish clearer guidelines. It is also important to study compliance with each specific standard within the bundle used during this project. Findings of inconsistency with a specific standard would help to guide future interventions and to ensure that radiologists maintain compliance with the established protocol over time.

Future research is also needed to confirm that we maintain compliance with these guidelines within our institution, past the 24-month period evaluated during this project. Additionally, work is needed to standardize clinical assessment procedures, as has begun for SLPs evaluating VFSSs with adults.^17,19^ Because this project only included one large pediatric institution, it is important to examine the results of these guidelines, and associated radiation exposure, after adoption by additional institutions.

## CONCLUSIONS

VFSSs are a vital component in the evaluation of the swallowing mechanism, including the assessment for aspiration. These examinations are performed in large numbers and require technical proficiency by a radiologist trained to understand the role of the VFSS in the assessment of pediatric swallowing. To our knowledge, there is not a published, standardized protocol to guide radiologists who perform pediatric VFSSs. Our interdisciplinary group of experienced professionals established a set of guidelines that emphasize the field of view, magnification, and pulse repetition rate for all pediatric VFSSs completed at our institution. Also, results from this QI project show that radiologists can successfully implement these guidelines into an academic practice utilizing a structured QI protocol. Future research projects should examine whether institutions can implement protocol changes for pediatric VFSSs such as those described in this article without increasing radiation exposure to these vulnerable patients.

## DISCLOSURE

The authors have no financial interest to declare in relation to the content of this article.

## Supplementary Material
