# Supplementary material for: Standardization of Radiologic Procedures for Pediatric Videofluoroscopic Swallow Studies: A Service-based Quality Improvement Initiative
Source: Pediatr Qual Saf. 2018 Dec 6;3(6):e123. doi: 10.1097/pq9.0000000000000123 (PMC6581479; doi:10.1097/pq9.0000000000000123)
Supplement: Supplementary file 1 [file pqs-3-e123-s001.pdf]

To be completed during your 1st week of Outpatient Fluoroscopy

- During your 1st week of Outpatient Fluoroscopy, please read the attached Standard VFSS 3 Easy Steps power point.
- Take the 3 question quiz. Please make sure to be descriptive in your answers.
- Have attending you are working with in outpatient fluoroscopy sign off on your quiz when you have finished.
- Turn Quiz only, into Program Coordinator at the end of your 1st week of Outpatient Fluoroscopy. Keep power point slides for further reference.

Thank you!

Standardized VFSS
3
Easy Steps

Step 1: Field of View (FOV)

Collimation borders

- Superior – Below orbits
- Inferior – Above C6 (excludes thyroid)
- Anterior – Lips
- Posterior – Spine

Step 2: Magnification

- Balance between radiation and anatomic detail
- Never use (3 X) magnification. 3 x magnification = 8 times radiation dose!
- Remember ALARA.
- Under 1 y.o. may require 2 x magnification. Over 1 y.o. should not require 2 x magnification

Step 3: Pulse Repetition Rate (PRR)

- 30 frames per second is standard of care. If less than 30 frames per second, may miss aspiration. This is well documented in literature.

- Our technologist are very good at setting correct PRR. With that being said, occasionally the image may be grainy. If this is the case please ask technologist to check PRR.

Review Questions
(Please return to Program Coordinator)

1. How many frames per second is the standard of care for the pulse repetition rate of a VFSS?

2. Please define the collimation borders for an appropriate F.O.V.?

3. True or False – When utilizing magnification, the radiation dose to the patient increases by the square of the ratio of the image intensifier diameters?

Resident Name: ________________________________

Faculty Signature:________________________________

Thank you!
